# Pulsed-field ablation-based pulmonary vein isolation: acute safety, efficacy and short-term follow-up in a multi-center real world scenario

**DOI:** 10.1007/s00392-022-02091-2

**Published:** 2022-09-22

**Authors:** Marc D. Lemoine, Thomas Fink, Celine Mencke, Ruben Schleberger, Ilaria My, Julius Obergassel, Leonard Bergau, Vanessa Sciacca, Laura Rottner, Julia Moser, Shinwan Kany, Fabian Moser, Paula Münkler, Leon Dinshaw, Paulus Kirchhof, Bruno Reissmann, Feifan Ouyang, Philipp Sommer, Christian Sohns, Andreas Rillig, Andreas Metzner

**Affiliations:** 1 Department of Cardiology, University Heart & Vascular Center Hamburg, University Medical Center Hamburg-Eppendorf, Martinistraße 52, 20246 Hamburg, Germany; 2 DZHK, Partner Site Hamburg, Kiel, Lübeck, Hamburg, Germany; 3 Clinic for Electrophysiology, Herz- Und Diabeteszentrum NRW, Ruhr-Universität Bochum, Bad Oeynhausen, Germany; 4 Institute of Cardiovascular Sciences, University of Birmingham, Birmingham, UK

**Keywords:** Atrial fibrillation, Catheter ablation, Pulsed-field ablation, Pulmonary vein isolation, Prospective observational study, real-world

## Abstract

**Purpose:**

Pulsed-field ablation (PFA) is a new energy source to achieve pulmonary vein isolation (PVI) by targeted electroporation of cardiomyocytes. Experimental and controlled clinical trial data suggest good efficacy of PFA-based PVI. We aimed to assess efficacy, safety and follow-up of PFA-based PVI in an early adopter routine care setting.

**Methods:**

Consecutive patients with symptomatic paroxysmal or persistent atrial fibrillation (AF) underwent PVI using the Farawave® PFA ablation catheter in conjunction with three-dimensional mapping at two German high-volume ablation centers. PVI was achieved by applying 8 PFA applications in each PV.

**Results:**

A total of 138 patients undergoing a first PVI (67 ± 12 years, 66% male, 62% persistent AF) were treated. PVI was achieved in all patients by deploying 4563 applications in 546 PVs (8.4 ± 1.0/PV). Disappearance of PV signals after the first application was demonstrated in 544/546 PVs (99.6%). More than eight PFA applications were performed in 29/546 PVs (6%) following adapted catheter positioning or due to reconnection as assessed during remapping. Mean procedure time was 78 ± 22 min including pre- and post PVI high-density voltage mapping. PFA catheter LA dwell-time was 23 ± 9 min. Total fluoroscopy time and dose area product were 16 ± 7 min and 505 [275;747] cGy*cm^2^. One pericardial tamponade (0.7%), one transient ST-elevation (0.7%) and three groin complications (2.2%) occurred. 1-year follow-up showed freedom of arrhythmia in 90% in patients with paroxysmal AF (*n* = 47) and 60% in patients with persistent AF (*n* = 82, *p* = 0.015).

**Conclusions:**

PFA-based PVI is acutely highly effective and associated with a beneficial safety and low recurrence rate.

**Graphical abstract:**

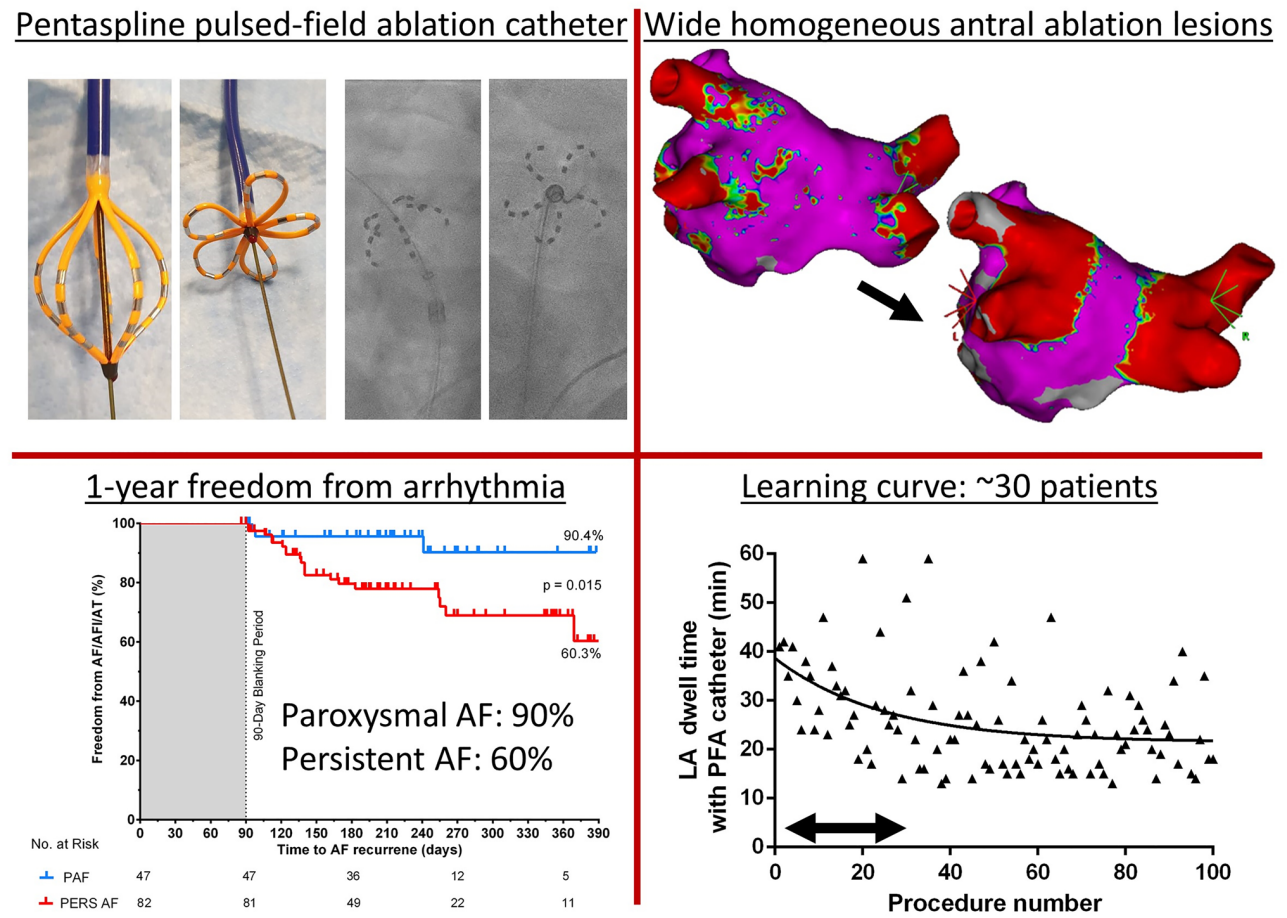

**Supplementary Information:**

The online version contains supplementary material available at 10.1007/s00392-022-02091-2.

## Introduction

Pulmonary vein isolation (PVI) is an important component of rhythm control therapy in patients with atrial fibrillation (AF). Durable and safe isolation of all PVs remains difficult [1] despite technical improvements including introduction of cryoballoon single shot devices [2], irrigated radiofrequency catheters, and contact force sensing ablation catheters [3]. In addition, unintended damage to other atrial or extracardiac structures can cause rare but severe complications, such as phrenic nerve palsy [4], PV stenosis [5], or atrio-esophageal fistula.

Pulsed-field ablation (PFA) is a nonthermal energy source in which electrical fields are used to disrupt the membrane of cardiomyocytes, whereas damage to other intra- and extracardiac anatomical structures, such as vessels, nerves or the esophagus is limited in experimental settings [6–9]. Known complications of thermal energy sources such as atrio-esophageal fistula, phrenic nerve palsy or PV stenosis have not been observed yet after PFA [10–12]. Based on these observations, PFA might have the potential to improve effectiveness of PVI with lower complication rates. Initial clinical observations in controlled trials appear to confirm the efficacy of PFA-based PVI results, showing PV reconnection in 4% of PVs (16% of patients) at 90 days and freedom from any atrial tachycardia in 85% [13], but more clinical information on the safety, efficacy and follow-up data of PFA-based PVI is needed.

Here, we report the periprocedural safety, efficacy and follow-up data of PFA-based PVI in two German high-volume centers.

## Methods

### Patient population

Data from consecutive patients with symptomatic paroxysmal or persistent AF who underwent PVI using the novel PFA ablation catheter (Farawave®, Farapulse Inc, Menlo Park, CA, USA) were analyzed. Exclusion criteria were prior PVI or left atrial (LA) ablation, LA diameter > 60 mm, severe valvular heart disease or contraindications to post-interventional oral anticoagulation. The study was performed in accordance with the Declaration of Helsinki of 2013, and it was designed as a prospective observational study approved by the ethics committee in Hamburg (NCT05521451, 2020-10066-BO) and Bad Oeynhausen (2019-563).

### Preprocedural management

Transesophageal echocardiography or cardiac computed tomography were performed to rule out LA thrombus prior to ablation. In patients on vitamin K antagonists, ablation was performed under therapeutic INR values of 2–3. Novel oral anticoagulants were skipped in the morning of the procedure. All patients provided written informed consent.

### Ablation procedure

The procedure was performed under deep sedation or general anesthesia with endotracheal intubation applying propofol, fentanyl and optional midazolam. After femoral venous access, a 7 F steerable decapolar catheter (Inquiry™, Abbott) was positioned in the coronary sinus. Following single transseptal puncture guided by fluoroscopy and using a modified Brockenbrough technique, a SL1 sheath (8.5 F, St. Jude Medical, MN, USA) was advanced into the LA. After transseptal puncture, intravenous heparin was administered to maintain an activated clotting time of >300 s. Selective PV angiography was performed to identify the individual PV ostium. Procedures in Bad Oeynhausen were performed after prior cardiac computer tomography or magnetic resonance imaging for three-dimensional (3D) integration (EnSite™NavX™and MediGuide™, Abbott). A 3D electroanatomical map of the LA was generated using a multipolar mapping catheter in conjunction with CARTO3 (Biosense Webster, Irvine, CA, USA) or the Ensite cardiac mapping system (NavX, Abbott, Inc.). Thereafter, the SL1 sheath was changed over-the-wire for a steerable sheath (Faradrive, 13 F inner and 16.8 F outer diameter). A 12 F multielectrode pentaspline PFA catheter (Farawave, Farapulse) was advanced over a soft, conventional J-tip wire (e.g., InQwire Guidewire J Tip-3 mm × 180 cm, Merit Medical). The PFA catheter size (diameter of 31 vs. 35 mm measured in fully deployed flower configuration) was chosen based on PV visualization by angiography and LA size. Preinterventional injection of 1 mg atropine were applied at operator’s discretion. After two applications in the basket configuration the pentaspline catheter was rotated one tenth of a rotation for another 2 applications in the same configuration. Thereafter 2 plus 2 applications were added in the flower configuration. Left common PVs were isolated by a similar approach receiving 16 applications (8 applications with the guidewire in the upper branch lower branch each). Generator output ranged from 1.8 to 2.0 kV per application during the course of the study with 5 biphasic and bipolar pulsed electrical fields of 200 ms duration each and 300 ms pause (2.5 s per application). PVI was defined as elimination of all PV signals and confirmed by entrance block using a circular mapping catheter. Remapping was performed to assess lesion formation. Other electrophysiological protocols performed after completion of PVI and remapping, e.g., programmed stimulation, slow-pathway modulation, or ablation at the cavo-tricuspid isthmus were not counted for procedure time of PVI.

### Endpoints

The primary endpoint was electrical PVI. Secondary endpoints included: PVI after single PFA application, acute PV reconnection, procedure-, LA dwell- and fluoroscopy times, dose area product, freedom from AF, atrial tachycardia or atrial flutter during 1-year follow up following a 90 days blanking period postablation, and procedure-related complications. The following complications were classified as major: pericardial effusion or tamponade, transient ischemic attack of stroke, atrioesophageal fistula, access site complications requiring surgical repair or blood transfusion. The following complications were classified as minor: Access site bleeding not requiring surgical or interventional therapy or blood transfusion.

### Postprocedural care

Following ablation all patients underwent transthoracic echocardiography to rule out pericardial effusion. All patients underwent a thorough clinical evaluation after the procedure and on the day of discharge, including a neurological assessment, to capture complications. All patients were treated with proton‐pump inhibitors for 6 weeks. Low molecular‐weight heparin was administered in patients on vitamin K antagonists in case of an INR < 2.0 until a therapeutic INR of 2 to 3 was reached. Novel oral anticoagulants were resumed 6 h post ablation. Anticoagulation was continued for at least 3 months, and thereafter based on the individual CHA_2_DS_2_‐VASc score. After the procedure antiarrhythmic drugs were stopped at the discretion of the operator.

### Follow-up

Patients were interviewed at 3, 6 and 12 months after the procedure concerning symptomatic recurrences. ECGs and Holter–ECGs were requested when in person follow-up was not conducted at the institution. Any episode of AF, atrial tachycardia (AT) or atrial flutter (AFl) as documented in 12-lead ECG or in Holter ECG > 30 s was considered a recurrence. Episodes that occurred during the first 90 days (blanking period) after the procedure were not considered recurrences.

### Statistical analysis

Continuous data are described as means and standard deviations, if the variables are normally distributed, or as medians, 25th and 75th percentiles otherwise. Categorical data are described with absolute and relative frequencies. All *p* values are two-sided and *p* < 0.05 is considered to indicate statistical significance. All calculations were performed with GraphPad Prism 6 or Microsoft Excel 365.

## Results

### Patient characteristics

**Fig. 1:**
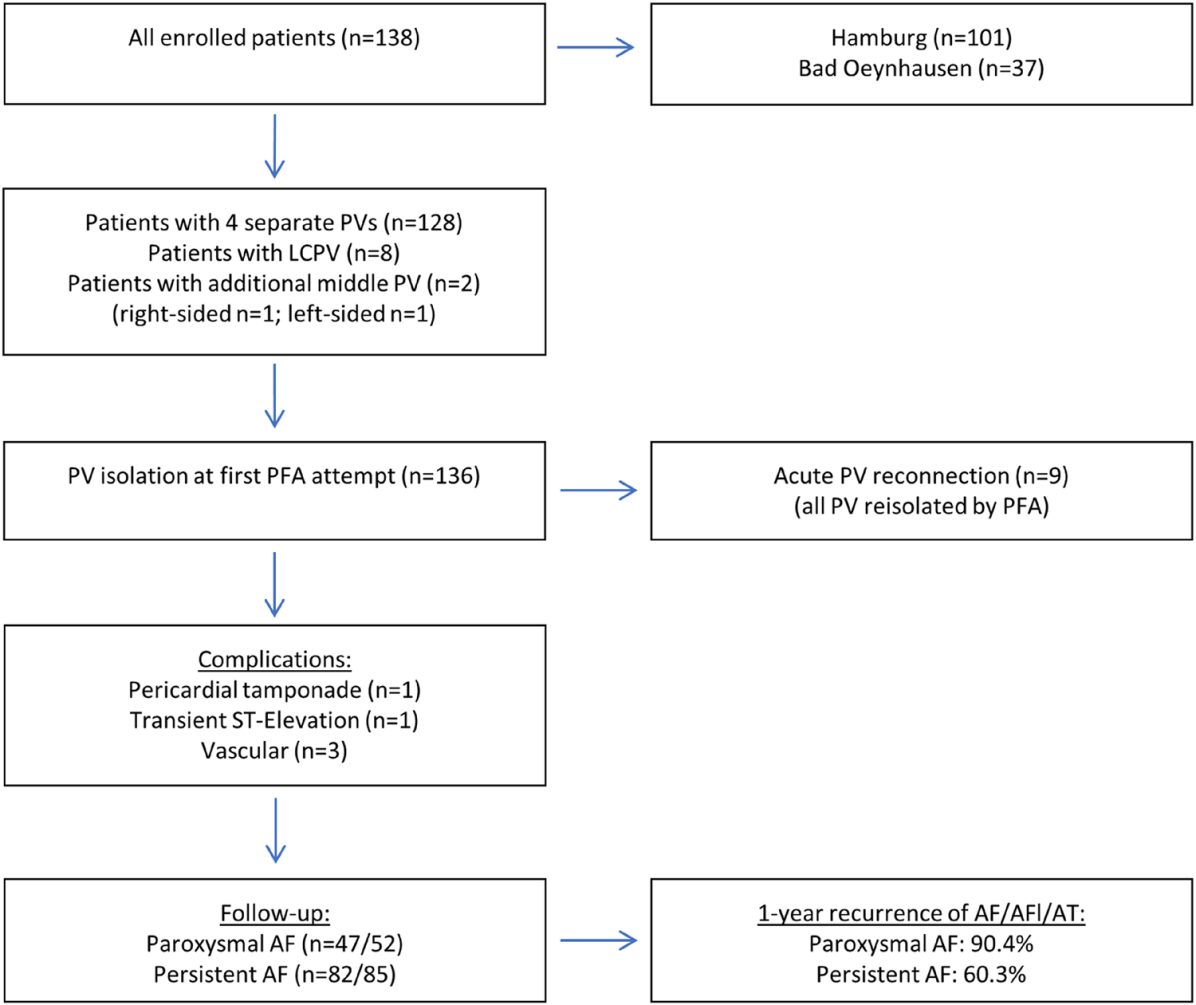
Flow chart of patients included in this prospective observational study (STROBE format). LC = left common, PFA = pulsed-field ablation, PV = pulmonary vein, AF = atrial fibrillation, AFl = atrial flutter, AT = atrial tachycardia

138 Patients with symptomatic paroxysmal (38%) or persistent AF (62%) were analyzed, 101 consecutive patients in Hamburg and 37 consecutive patients in Bad Oeynhausen (Fig. 1). Mean age was 67 ± 12 years with male predominance (66%), CHA_2_DS_2_–VASc Score of 2.7 ± 1.7, and with moderate LA enlargement (diameter 43 ± 5 mm, volume index 37 ± 13 ml/m^2^). Detailed patient characteristics are given in Table 1.

**Table 1:** Baseline patient characteristics (n = 138)

Variable	Statistics
Age (years)	67 ± 12
Male, *n* (%)	91/138 (65.9)
BMI (kg/m^2^)	28 ± 6
Paroxysmal atrial fibrillation, *n* (%)	52/138 (37.7)
LA diameter (mm)	43 ± 5
LA volume (ml)	81 ± 37
LA volume index (ml/m^2^)	37 ± 13
LVEF (%)	52 ± 10
LVEF ≥ 50%	106/138 (76.8)
LVEF 40–49%	15/138 (10.9)
LVEF 30–39%	10/138 (7.2)
LVEF < 30%	7/138 (5.1)
CHA_2_DS_2_–VASc Score	2.6 ± 1.7
Congestive heart failure, *n* (%)	34/138 (24.6)
Arterial hypertension, *n* (%)	90/138 (65.2)
Diabetes mellitus, *n* (%)	23/138 (16.7)
Vascular disease, *n* (%)	26/138 (18.8)
Stroke/TIA, *n* (%)	6/138 (4.3)
Antiarrhythmic drugs at baseline, *n* (%)	26/138 (18.8)
Betablocker, *n* (%)	110/138 (79.7)
Flecainide or Propafenone, *n* (%)	11/138 (8.0)
Amiodarone, (%)	15/138 (10.9)
Aorto-coronary bypass, *n* (%)	4/138 (2.9)
Valve-replacement/reconstruction, *n* (%)	7/138 (5.1)
Mitral-clipping, *n* (%)	2/138 (1.4)
Pacemaker/Intracardiac defibrillator, *n* (%)	6/138 (4.3)

Continuous data are summarized as means ± standard deviations. Categorical data are presented as n (%)

*BMI* = body mass index, *LA* = left atrial, *LVEF* = left ventricular ejection fraction

### Procedural parameters

**Fig. 2:**
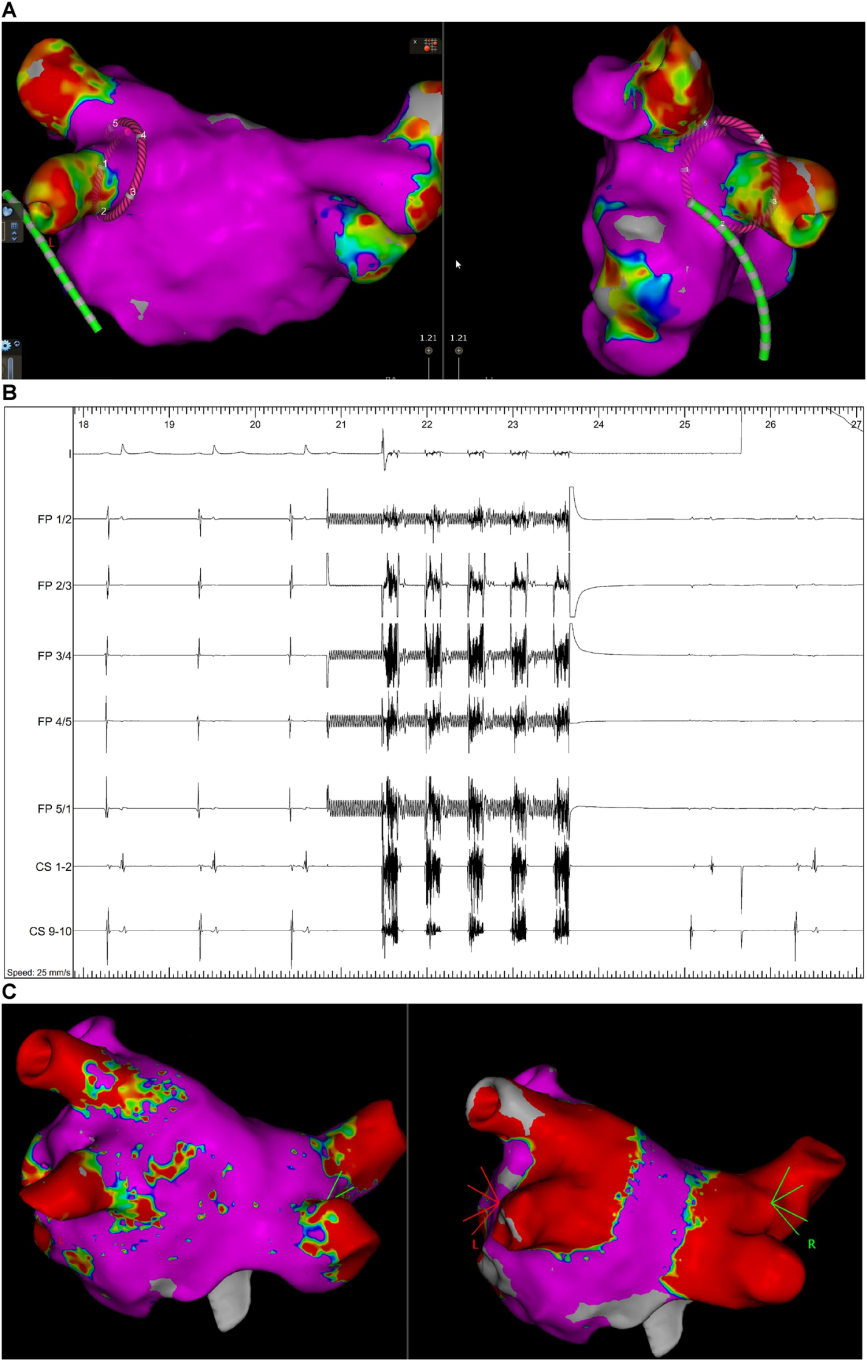
**A** Example of pulsed-field ablation catheter visualization in CARTO3 (Biosense Webster) at the LIPV in posterior–anterior (left) and left lateral projection (right). **B** Example of disappearance of pulmonary vein signals at the left superior pulmonary vein after the first pulsed-field application. **C** Three-dimensional electroanatomic voltage map of the left atrium in postero-anterior before (left) and after PFA-based pulmonary vein isolation (right)

**Fig. 3:**
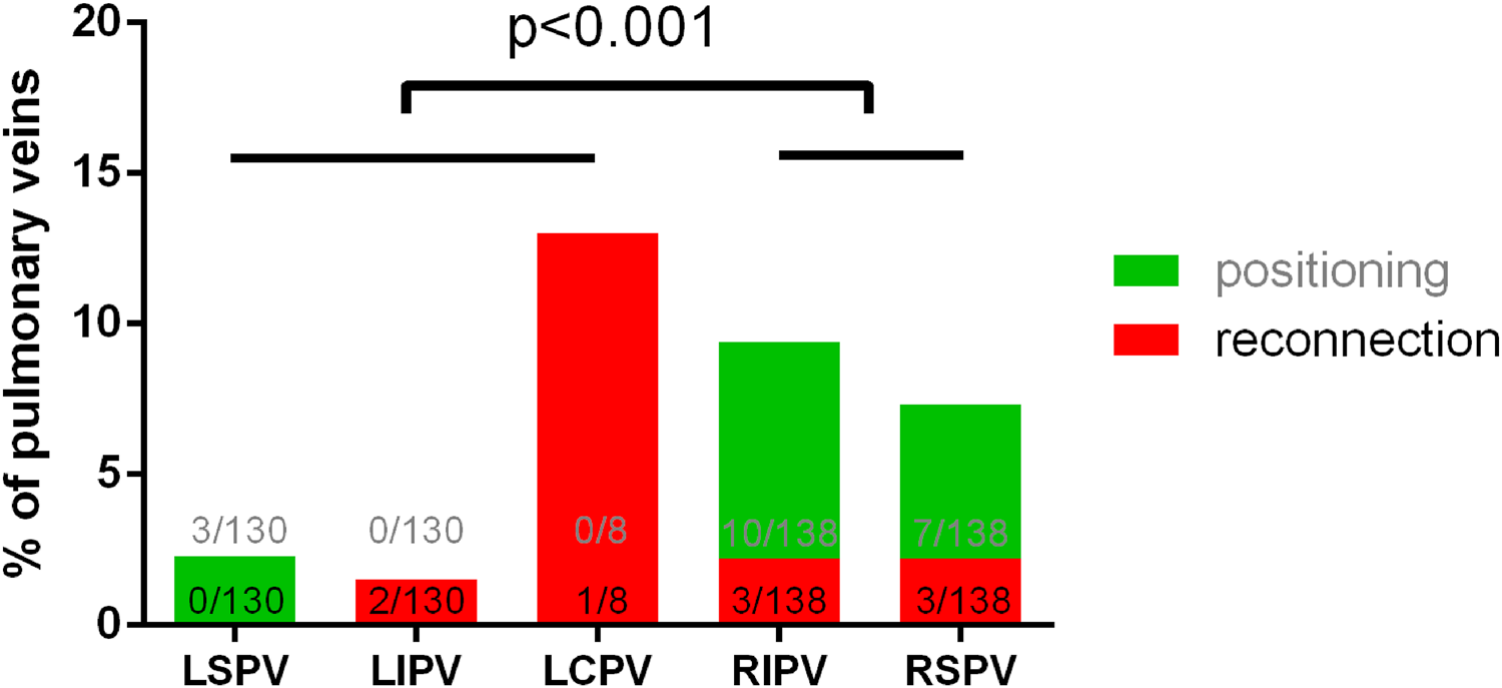
Quantification of pulsed-field applications additional to the recommended 8 applications per pulmonary vein. Green represents additional application due to positioning on operator’s discretion. Red represents additional applications due to reconnection of pulmonary vein. LSPV = left superior PV, LIPV = left inferior PV, LC = left common, PFA = pulsed-field ablation, PV = pulmonary vein, RIPV = right inferior PV; RSPV = right superior PV

**Fig. 4:**
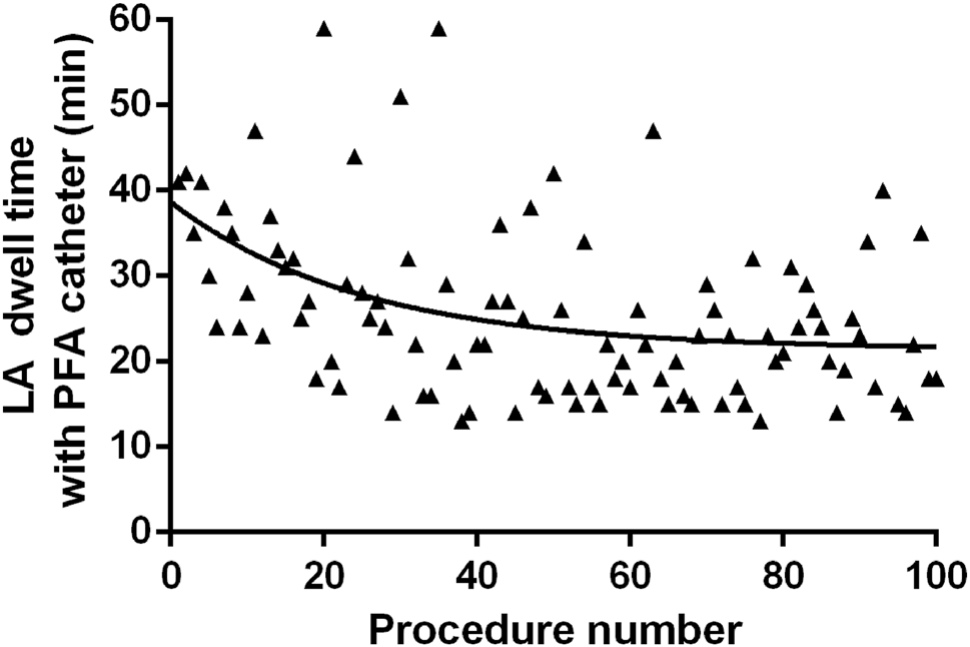
Single-center learning curve illustrated by PFA catheter LA dwell time during the first 101 cases in Hamburg. Each dot shows LA dwell time of the PFA catheter, the line is a best fitted exponential graph

Mean procedure time (defined as time from femoral access until sheath removal or begin of a secondary ablation) was 78 ± 22 min including pre- (15 ± 8 min) and post-PVI (11 ± 5 min) voltage mapping time (Fig. 2C). Mean LA dwell time was 60 ± 20 min, PFA catheter dwell time was 24 ± 10 min (Table 2). The mean total number of PFA-applications was 33 ± 3/patient corresponding to a PVI mean ablation time of 83 ± 8 s/patient. Most additional applications were applied at right-sided PVs (23/277) vs. left-sided PVs (6/269; *p* < 0.001, Fig. 3). During the first 101 cases in Hamburg the PFA catheter dwell time was reduced from 32 ± 10 min (first 30 pts) to 23 ± 8 min (*p* < 0.001; 61–101st patient, Fig. 4), which was not significantly different from patient 31–61st (24 ± 10 min, *p* = 0.951). This reflects a learning curve reaching a plateau after approximately 30 procedures**.**

**Table 2:** General procedural data (n = 138)

Variable	Statistics
Procedure time (min)	78 ± 22
Total LA dwell time (min)	60 ± 20
Index mapping (min)	15 ± 8
PFA Catheter LA dwell time (min)	23 ± 9
Remapping (min)	11 ± 5
Total fluoroscopy time (min)	16 ± 7
PFA Catheter LA fluoroscopy time (min)	9 ± 5
Total area dose product (cGy*cm^2^)	505 [275;746]
PFA Catheter LA area dose product (cGy*cm^2^)	315 ± 391
Mean number of applications	33 ± 3
LSPV PFA-applications	8.1 ± 0.3
LIPV PFA-applications	8.1 ± 1.0
LCPV PFA-applications	16.1 ± 1.62
RSPV PFA-applications	8.5 ± 1.9
RIPV PFA-applications	8.2 ± 1.4
PV isolation with 1. Application, *n* (%)	544/546 (99.6)
Time to isolate RPV (min)	11 ± 7
Time to isolate LPV (min)	10 ± 4
Non-PV PFA applications, *n* (%)	
LA anterior wall, *n* (%)	2/138 (1.4)
Superior vena cava, *n* (%)	1/138 (0.7)
Cavotriscupid isthmus ablation, *n* (%)	4/138 (2.9)
RF-touch-up for PVI, *n* (%)	0/138 (0)
RF-touch-up for additional ablation	
Cavotriscupid isthmus ablation, *n* (%)	9/138 (6.5)
Slow pathway ablation, *n* (%)	1/138 (0.7)
Complications—major	1/138 (0.7)
Cardiac tamponade (venous), *n* (%)	1/138 (0.7)
Complications—minor	4/138 (2.9)
Transient ST-Elevation, *n* (%)	1/138 (0.7)
Vascular complication (groin) with conservative treatment, *n* (%)	3/138 (2.2)

Continuous data are summarized as means ± standard deviations or as medians, 25^th^ and 75^th^ percentiles otherwise. Categorical data are presented as n (%)

*LA* = left atrial, *LC* = left common, *LI* = left inferior, *LS* = left superior, *PFA* = pulsed-field ablation, *PV* = pulmonary vein, *PVI* = PV isolation, *RI* = right inferior, *RPV* = right-sided PV, *RS* = right superior

### Acute efficacy

**Fig. 5:**
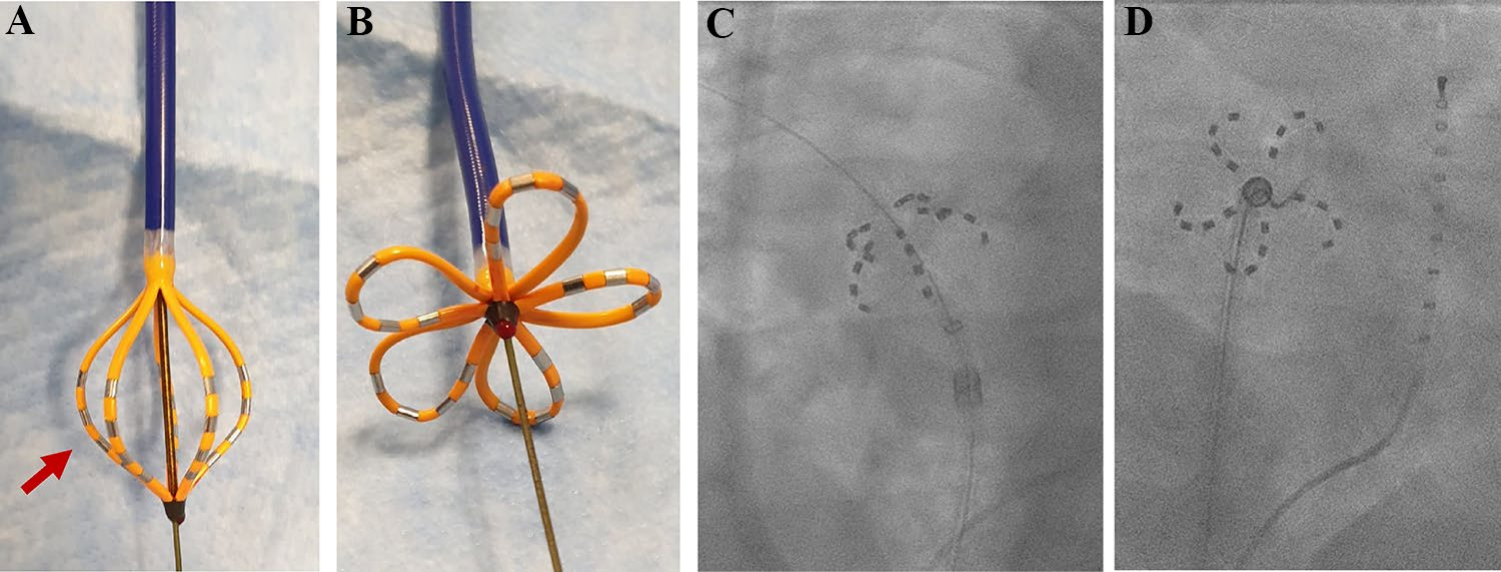
Pulsed-field ablation catheter (FARAPULSE) in its basket (**A**) and flower (**B**) configuration. Corresponding fluoroscopic views of the pulsed-field ablation catheter at a right superior pulmonary vein in basket (**C**, right anterior oblique 30°) and flower (**D**, left anterior oblique 40°) configuration. Red arrow indicates subequatorial electrode exemplary for one spline, which can be visualized in 3D mapping systems (Fig. 3)

Successful electrical PVI was achieved in 546/546 PVs (100%), including 8 left common PVs and 1 left and 1 right middle PV. Of note, PV signals recorded on the pentaspline PFA catheter disappeared after the first application (2.5 s) in 544/546 PVs (99.5%, for example, see Fig. 2B). A total of 515/546 PVs (94.3%) was treated by 8 applications (4 applications each in the basket and flower configuration, Fig. 5) according to the current recommendations. In 17/138 patients (12.3%) and 20/546 PVs (3.7%) additional applications were deployed due to adapted positioning of the catheter at the respective PVs (Table 3). Acute PV reconnection was observed during remapping in 9/138 patients (7%) and in 10/546 PVs (1.8%, Fig. 3, Table 3). All these PVs were re-isolated by additional PFA applications in adapted catheter positions. No RF touch-up was necessary. PFA induced wide antral circumferential lesions around the ipsilateral PVs (Fig. 2), even in left common PVs (S-Fig. 1). In a subset of post-PVI voltage maps (*n* = 33), posterior distance between left and right low-voltage areas was reduced from 69 ± 12 mm prior ablation to 25 ± 10 mm (*p* < 0.001) after PVI at superior PVs and from 61 ± 10 mm to 25 ± 10 mm (*p* < 0.001) at inferior PVs with a remaining non-ablated LA posterior wall area of 11.9 ± 4.7 cm^2^.

**Table 3:** Detailed analysis of pulsed-field applications and lesion characterization

Variable	Statistics
Procedures with regular 32 applications, *n* (%)	121/138 (87.7)
Procedures with additional PV applications, *n* (%)	17/138 (12.3)
Additional applications before remapping due to positioning/operator discretion, *n* (%)	20/546 (3.7)
- LSPV, *n* (%)	3/130 (2.3)
- LIPV, *n* (%)	0/130 (0)
- LCPV, *n* (%)	0/8 (0)
- RIPV, *n* (%)	10/138 (7.2)
- RSPV, *n* (%)	7/138 (5.1)
Additional applications after remapping due to reconnection, *n* (%)	9/546 (1.6)
- LSPV, *n* (%)	0/130 (0)
- LIPV, *n* (%)	2/130 (1.5)
- LCPV, *n* (%)	1/8 (12.5)
- RIPV, *n* (%)	3/138 (2.2)
- RSPV, *n* (%)	3/138 (2.2)
Lesion characterization (*n* = 32)	
- LA volume (incl. PVs)	165 ± 33
- Number of mapping points	3662 ± 1977
- Distance of PV low-voltage areas (superior)	
Before PVI (mm)	69 ± 12
After PVI (mm)	25 ± 10
- Distance of PV low-voltage areas (inferior)	
Before PVI (mm)	61 ± 10
After PVI (mm)	25 ± 10
- Non-ablated LA posterior wall area (cm^2^)	11.9 ± 4.7

Continuous data are summarized as means ± standard deviations. Categorical data are presented as n (%)

*LC* = left common, *LI* = left inferior, *LS* = left superior, *PV* = pulmonary vein; *PVI* = PV isolation, *RI* = right inferior, *RS* = right superior

Phrenic capture during PFA pulses was observed in 90/93 (96.8%) patients and was more frequently seen along the right than left PVs (88/93 vs. 30/93, *p* < 0.001) and more often at the superior vs. inferior PVs (85/93 vs. 75/93, *p* = 0.035). In detail, phrenic nerve capture occurred at 83/93 RSPVs (89.2%), 71/93 RIPVs (76.3%), 25/93 LSPVs (26.9%) and 15/93 LIPVs (16.1%). Atropine was administered in a total of 88/138 patients to reduce vagally induced bradycardia due to sinus pause (S-Fig. 2) or atrioventricular block (S-Fig. 3). 4/138 patients received atropine after previous distinct vagal reactions following first PFA pulses. Asystole was much shorter in patients with previous atropine administration (2.0 ± 0.7 s, *n* = 10) than without (5.9 ± 3.3 s, n = 10, p = 0.018). 49/138 patients did not receive atropine due to the operator’s discretion or due to implanted pacemakers with a ventricular lead or due to the ability to pace the ventricle via the coronary sinus catheter.

### Integration in mapping system

The PFA pentaspline catheter could be visualized within the 3D map with both mapping systems (CARTO3, Bisosense Webster, Fig. 2 and EnSite™NavX™, Abbott, S-Fig. 1), helping to navigate the catheter. The subequatorial electrode of each spline (third electrode) could be connected to the mapping system (Fig. 5), resulting in a ring in the 3D map (Fig. 2A), representing the external dimension of the catheter in basket or flower configuration. During PFA pulses it is recommended to pause the catheter visualization. When visualization was not paused, the catheter disappeared during PFA pulses and re-appeared within a few seconds afterward, but in few cases 3D mapping matrix was lost. With the Ensite mapping system, the pentaspline PFA catheter could also be used to create a 3D voltage map of the LA recorded by 5-electrodes in total, although mapping resolution was reduced compared to dedicated mapping catheters.

### Non-PV applications

Two patients developed an atrial tachycardia during the procedure and according to entrainment maneuvers the lateral anterior wall was involved in the circuit. PFA applications (4 applications in basket configuration in the first patient and 8 applications, 4 in basket- and 4 in flower configuration, in the second patient) at the anterior wall terminated the tachycardias. However, bidirectional conduction block was not assessed. In one patient with persistent AF, the posterior wall was isolated by 4 applications in flower configuration.

One patient with persistent AF suffered from repetitive onset of AF after multiple electrocardioversions after PFA-based PVI. The trigger origin was identified within the superior vena cava (SVC). The PFA catheter was then placed into the SVC and 4 PFA applications were conducted. During these applications sinus arrest occurred and after 5 min of atrial pacing sinus rhythm returned. After SVC ablation AF did not re-occur. However, isolation of SVC was not assessed.

In 4/138 patients (2.9%) bidirectional block of the CTI was performed after PVI with PFA by 9 ± 2 applications and in 9/138 patients (6.5%) by RF ablation. One patient received slow-pathway modulation by RF after induction of atrioventricular nodal reentry tachycardia.

### Periprocedural complications

Periprocedural complications were one cardiac tamponade requiring pericardiocentesis (blood gas analysis suggested venous bleeding, potentially indicating an association with a difficult insertion of the CS-catheter in this procedure), one patient experienced transient ST-elevation and concomitant atrioventricular-block for 3 min which resolved spontaneously and three minor groin site complications which were treated conservatively. One patient described minor neurological missperceptions and underwent cerebral MRI without any pathological signs, while symptoms disappeared within hours. No other adverse events occurred.

### Follow-up

**Fig. 6:**
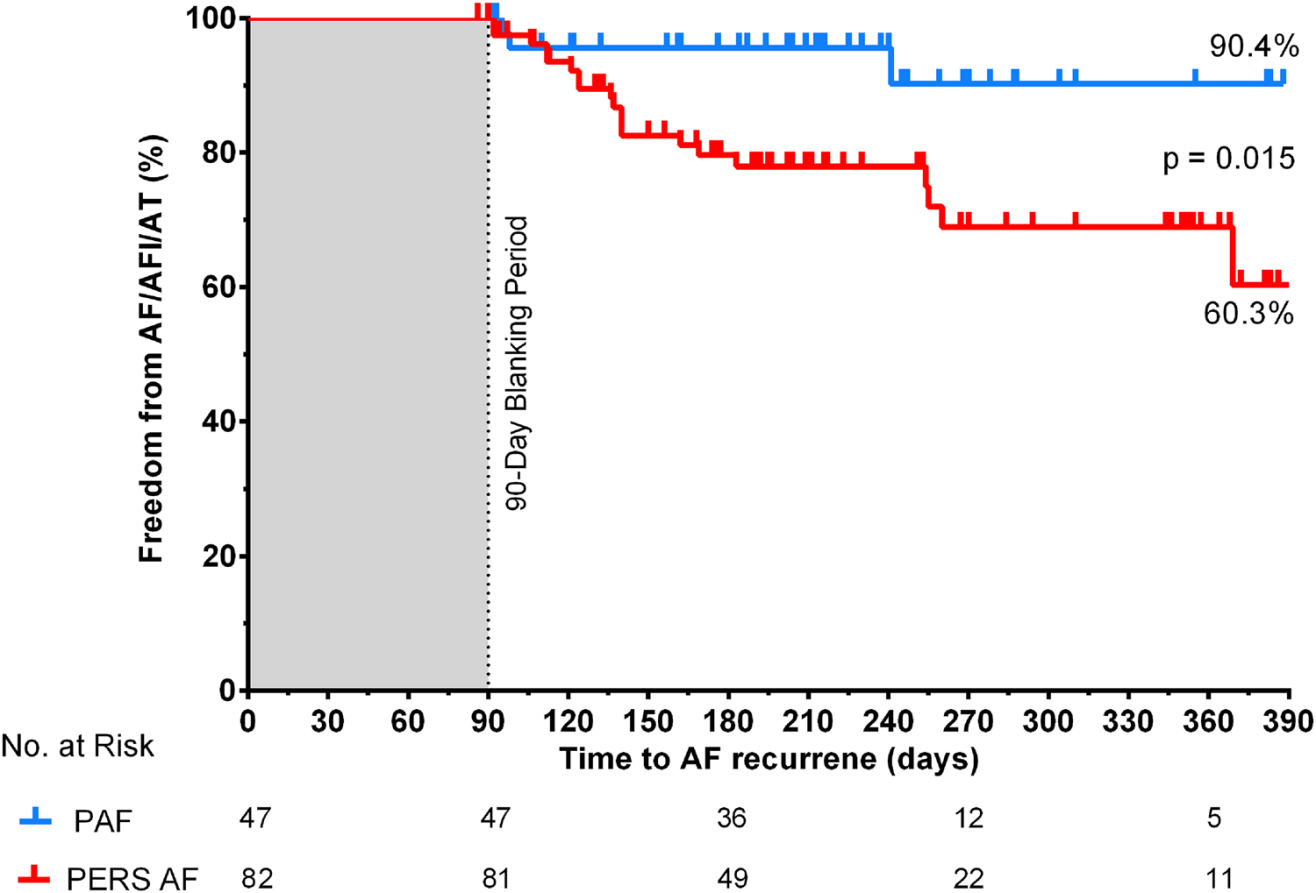
Kaplan–Meier curve showing freedom from AF/AFL or AT in paroxysmal (blue) and persistent (red) atrial fibrillation after initial pulmonary vein isolation by pulsed-field ablation during 1-year follow-up. AFl indicates atrial flutter; AF, atrial fibrillation and AT, atrial tachycardia

129 of 137 (94%) patients could be included into follow-up analysis with a mean follow-up time of 249 ± 90 days after PVI. The single procedure Kaplan–Meier estimates for freedom from AF, AFl or AT was 60 ± 10% for patients with persistent AF (*n* = 82) vs. 90 ± 6% in patients with paroxysmal AF (*n* = 47, *p* = 0.015, Mantel–Cox test, Fig. 6). At last follow-up, class I or III antiarrhythmic drug therapy was continued in 2.4% patients with persistent AF and in 2.1% with paroxysmal AF.

## Discussion

To the best of our knowledge, this study is the largest multi-center report in routine care evaluating acute efficacy, periprocedural safety and 1-year follow-up of PFA-based PVI system in patients with AF.

Main findings are:

1. PFA-based PVI achieves complete acute isolation of PV (546 of 546 PV),
2. acute complication rates are low (1/138 patients, 0.7%),
3. the PFA catheter can be visualized using commonly used mapping systems,
4. the system provides a short learning curve and a short PFA catheter LA dwell time,
5. atropine reduced the duration of post-PFA asystole and heart block,
6. freedom of AF/AT/AF was 90% for paroxysmal AF and 60% for persistent AF patients during 1-year follow-up.

### Efficacy of PFA-based PVI

In the current study we demonstrated that PVI by PFA and the Farawave catheter could be successfully performed in a total of 138 patients and in two independent German high-volume centers. Ablation was characterized by short procedure duration and ultrashort PVI ablation times when compared to reports of established systems, such as RF in conjunction with 3D mapping [4, 14]. In comparison to other PFA single-shot devices integrated in a circular catheter [15], the pentaspline catheter (basket/flower configuration) seems to have shorter procedure and LA dwell times, probably due to facilitated positioning. Total procedure times without 3D mapping would achieve < 60 min in this series. The combination of PFA catheter ablation and different 3D mapping platforms was easy and safe at both centers. Thus, the PFA-based system can be used for PVI [16, 17] including to achieve the clinical benefit of early rhythm control therapy [18] and in view of the superiority of ablation over drug-based treatment strategies regarding freedom from AF [19, 20].

In our “all comer” cohort we did not find any hint that PFA–PVI might be less successful in patients with advanced atriopathy. Nevertheless, similar to other energy-forms of PVI, non-PV trigger might jeopardize clinical success of PVI in patients with advanced atriopathy.

The rather short learning curve regarding PFA catheter LA dwell time demonstrated easy adoption of the technology in daily practice even when performed by multiple operators, reaching mean PFA LA dwell times of 23 min, which is even lower than previously reported for the pentaspline PFA catheter [13].

Comparable to previously published data from Reddy et al.[21], the intraprocedural PVI durability as assessed by final remapping was high and demonstrated in 98.4% PVs. PVs with electrical reconnection could be finally isolated with additional applications in adapted catheter positionings by, e.g., placing the guide wire to another PV branch or by giving the PFA catheter a more anterior orientation in the flower configuration.

### Safety

The safety profile with a single pericardial tamponade as the only major complication, and no TIA/stroke or phrenic nerve paralysis is consistent with previously published data [13]. Whether lack of esophageal or neuronal damage is due to the specifics of the PFA energy, or due to chance, will need further evaluation in independent data sets. Nevertheless, access to the LA via an 18 F sheath might bear a risk for air embolism, which might have been the cause of the observed transient ST-elevation and concomitant AV-block.

### Follow-up

Clinical success of PVI depends on PV durability and non-PV triggers. PV durability showed large heterogeneity in remapping studies after PVI with thermal energy sources (12–79% of patients) [1] and 84% in one remapping trial using the latest waveform generation for PFA [13]. Non-PV-triggers may depend on left ventricular function [22], LA enlargement, degree of fibrosis or sex [23]. Freedom from AF, AFl or AT in patients with paroxysmal AF showed comparable rates in this trial (90 ± 6%) as previously reported (85 ± 5%) [13]. To our knowledge, we report here for the first time 1-year follow-up data of patients with persistent AF (freedom of AF, AFl and AT of 60 ± 10%), which seems comparable to previous reports on thermal ablation [24].

### Technical considerations

Isolation of the RIPV was difficult in some patients: all 7 procedures with PFA catheter dwell times > 45 min (Fig. 4) were associated with difficulties in PFA catheter positioning along the respective RIPVs. In general, the right PVs needed more frequently additional PFA applications than left PV (Table 2). In the experience of the teams in Hamburg and Bad Oeynhausen, targeting an anterior transseptal puncture seems to facilitate positioning of the PFA catheter basket configuration with equal spline spacing.

We investigated systematically the effect of atropine on post-application vagal reaction after PFA pulses. Atropine reduced the duration of asystole and heart block as reported in single cases [25].

Treatment of non-PV-dependent atrial arrhythmias needs further investigation with regard to feasibility, efficacy and safety. In a recent publication coronary spasm after PFA application at the mitral isthmus was reported [26]. To the best of our knowledge, we report for the first time the PFA application at the SVC. In contrast to previous reports in swine [27], we observed a sinus pause of several minutes, which might be a direct effect on the sinus node due to its anatomical proximity or an indirect effect due to a vagal reaction. Further assessment of this potential treatment option is warranted.

### Limitations

The current study gained important insights into real-world performance data using the novel Farapulse PFA ablation system in a clinical multicenter investigation. However, the current study is an observational, prospective, non-randomized analysis in two high-volume centers and in a limited number of patients.

### Conclusions

In a multi-center real world setting, PVI by PFA is acutely highly effective, associated with a beneficial safety profile and a low recurrence rate.

## Supplementary Information

Below is the link to the electronic supplementary material.

Supplementary file1 (DOCX 3915 kb)

## Abbreviations

3D: Three-dimensional
AF: Atrial fibrillation
CTI: Cavo-tricuspid isthmus ablation
LA: Left atrium
LCPV: Left common pulmonary vein
LSPV: Left superior pulmonary vein
LIPV: Left inferior pulmonary vein
PFA: Pulsed-field ablation
PVI: Pulmonary vein isolation
PV: Pulmonary vein
RIPV: Right inferior pulmonary vein
RSPV: Right superior pulmonary vein
SVC: Superior vena cava
