# Supplementary material for: Pulsed-field ablation-based pulmonary vein isolation: acute safety, efficacy and short-term follow-up in a multi-center real world scenario
Source: Clin Res Cardiol. 2022 Sep 22;112(6):795–806. doi: 10.1007/s00392-022-02091-2 (PMC10241704; doi:10.1007/s00392-022-02091-2)
Supplement: Supplementary file 1 — Supplementary file1 (DOCX 3915 kb) [file 392_2022_2091_MOESM1_ESM.docx]

**Supplemental Material**

**
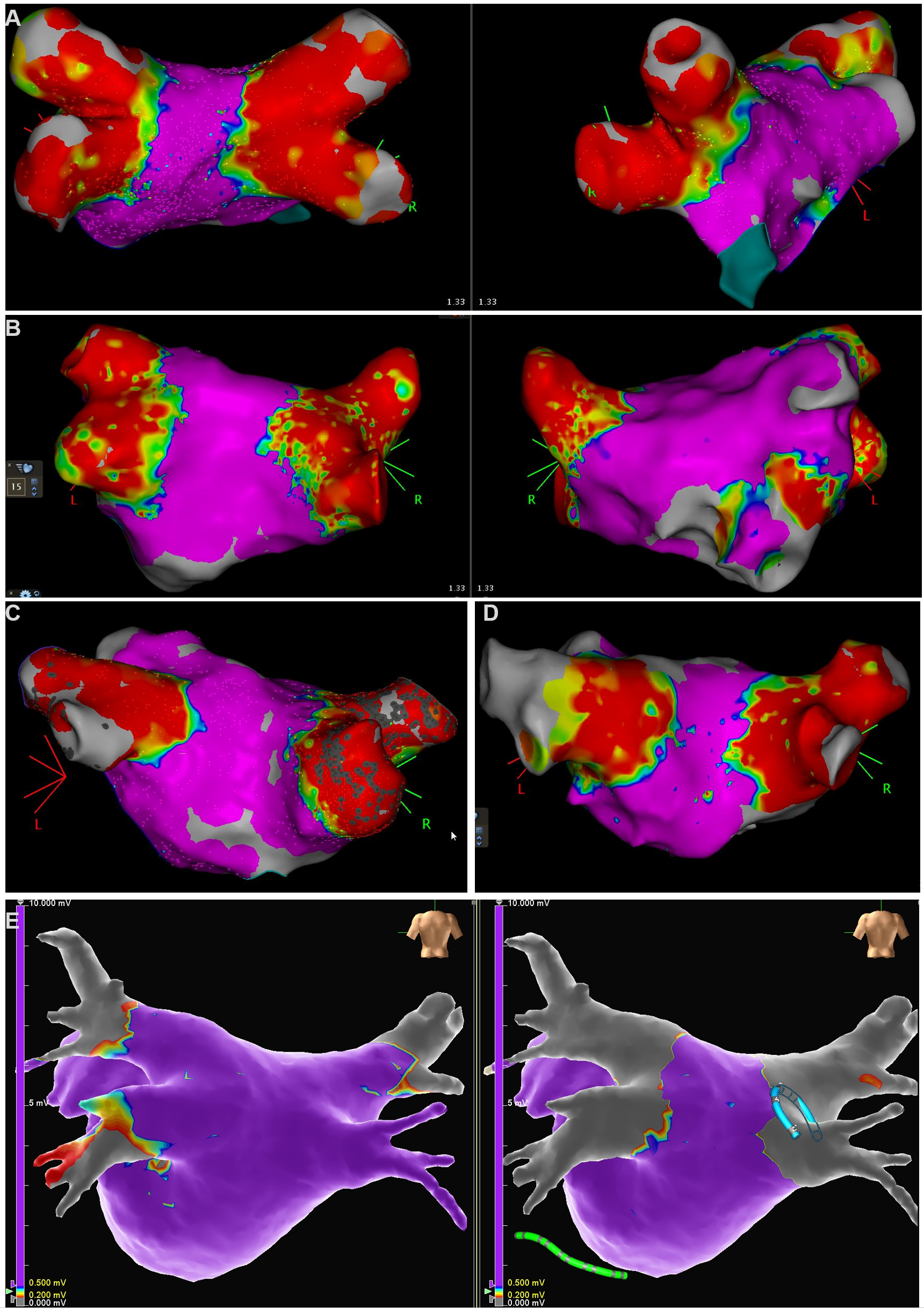
**

**Supplemental-Figure 1.** Other examples of left atrial voltage maps after PVI with PFA. (A) shows wide antral lesion in posterior-anterior (left panel) and right lateral view (right panel). (B) shows wide antral lesion in posterior-anterior (left panel) and anterior-posterior view (right panel). (C) and (D) show two examples of antral lesions around left common pulmonary veins. (E) shows left atrial voltage maps before (left) and after (right) PFA based-PVI and catheter visualization in (EnSite™NavX™, Abbott).

**A**


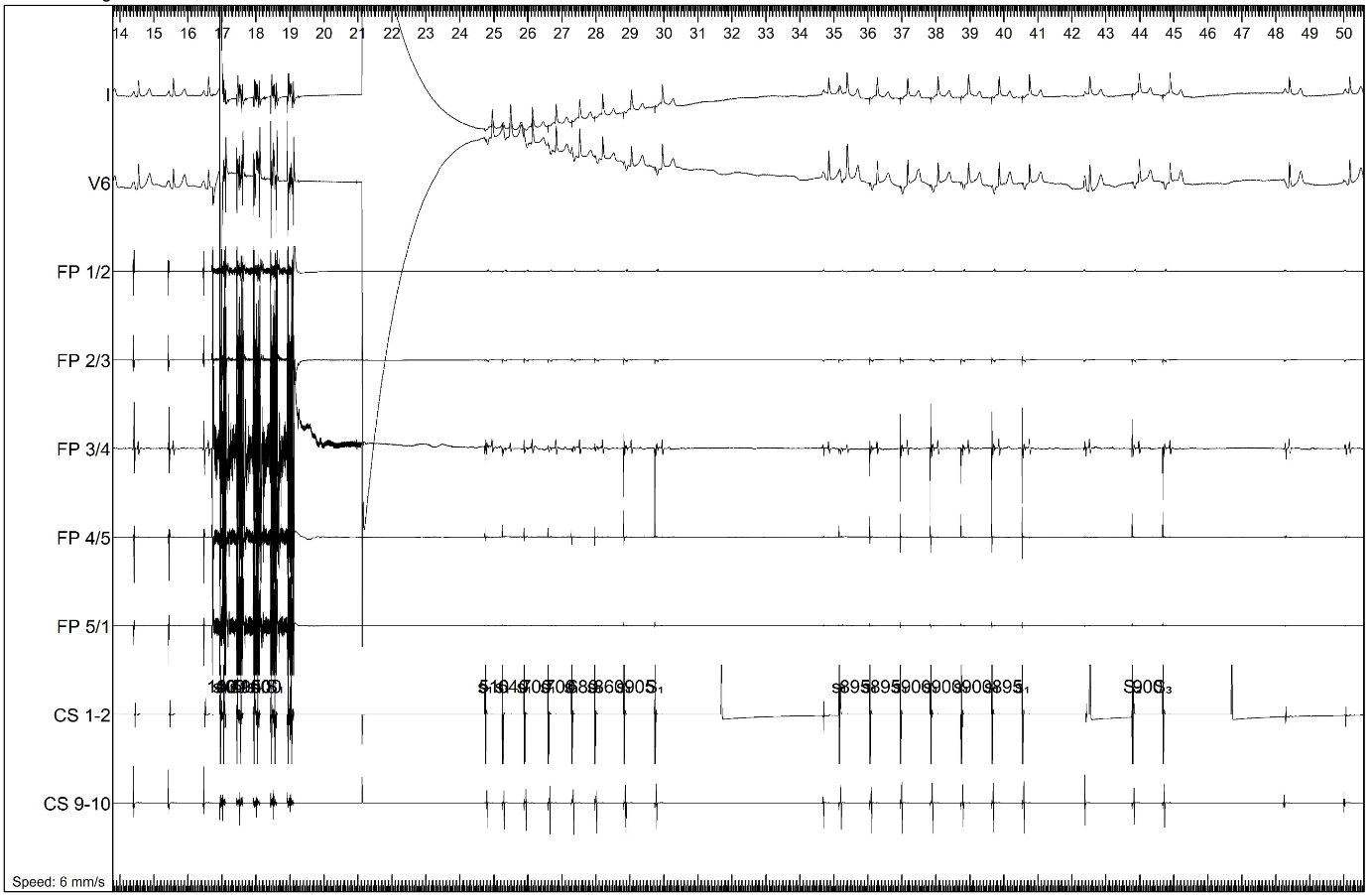


**B**


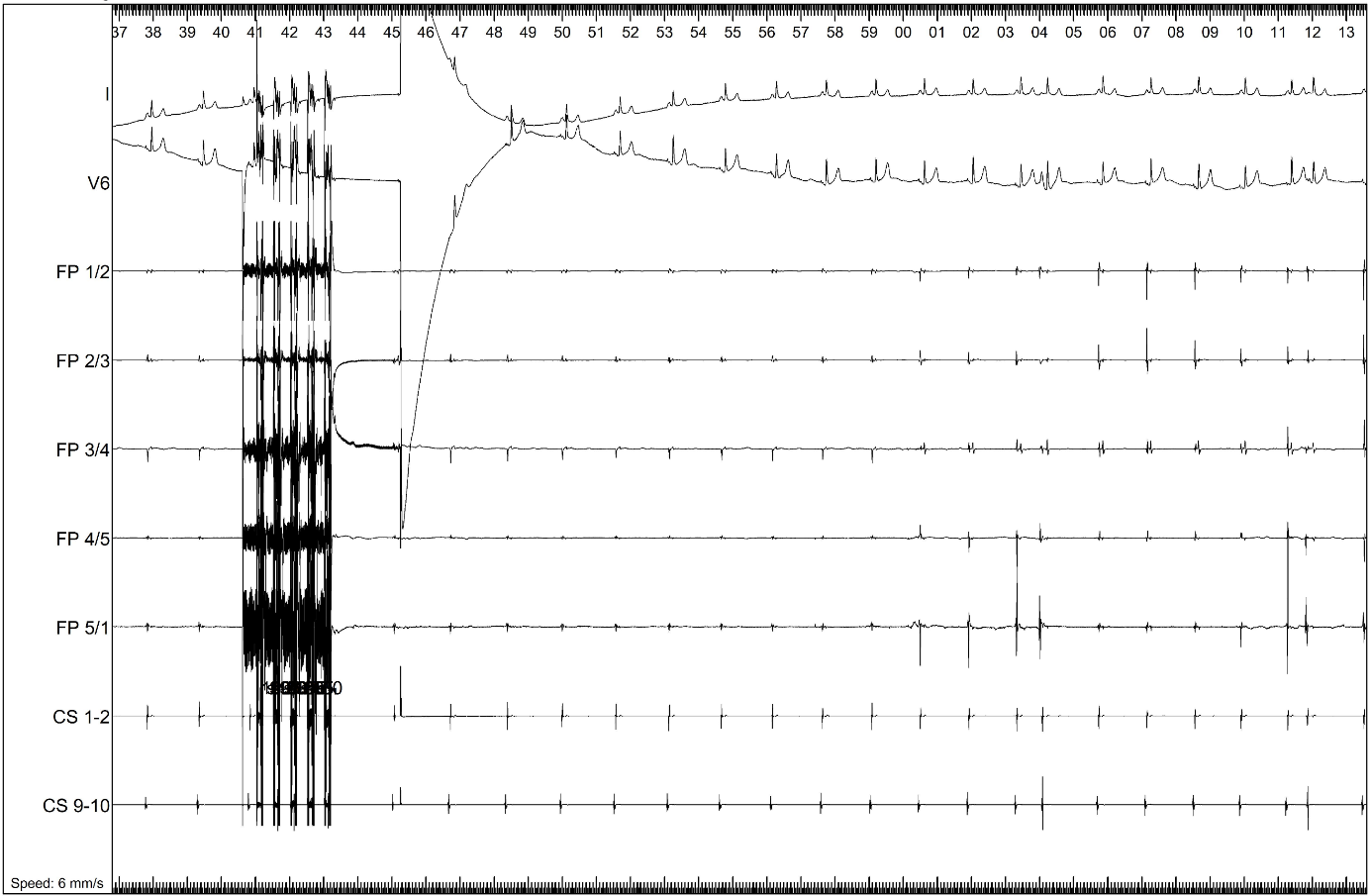


**Supplemental-Figure 2.** Example of PFA pulses at the left superior pulmonary vein followed by a sinus pause without previous administration of atropine. Atrial pacing was performed via coronary sinus catheter for 25 s, before sinus function returned after 30 s. (B) Example of PFA pulses at the left superior pulmonary vein of the same patients as in panel (A) but with previous administration of atropine leading to no relevant sinus pause.


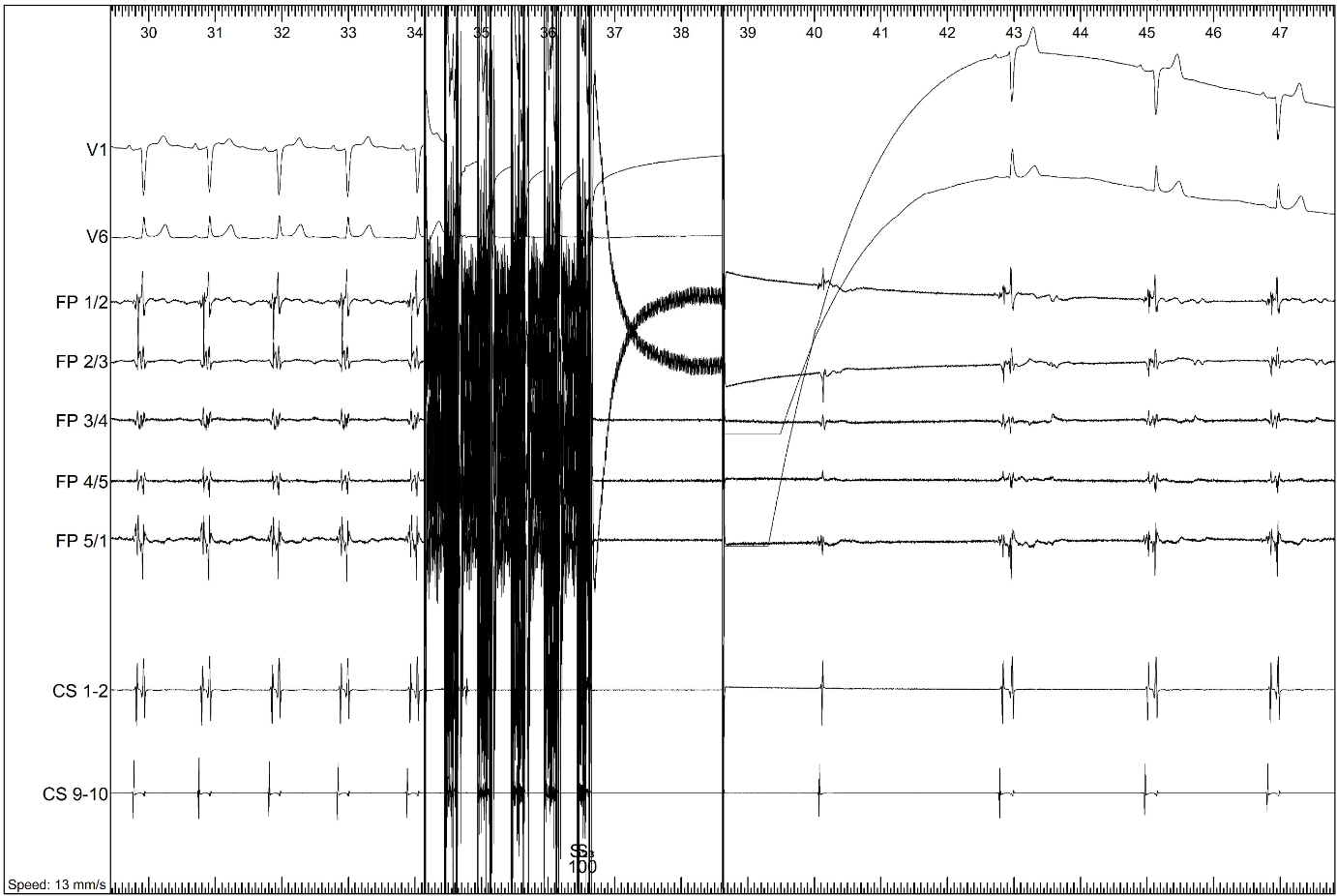


**Supplemental-Figure 3.** Example of PFA pulses at the left superior pulmonary vein followed by a sinus pause and atrioventricular block for >4 s without previous application of atropine.
